# The Nature of the Cancerous Process1Abstract of a paper read before the Surgical Section of the American Medical Association, at St. Paul, June 5, 1901.

**Published:** 1901-07

**Authors:** Roswell Park

**Affiliations:** Professor of surgery, Medical Department of the University of Buffalo


### BUFFALO MEDICAL JOURNAL.

VOL. XL.—LVI. JULY, 1901. No. 12.

### Original Communications.

### THE NATURE OF THE CANCEROUS PROCESS.[1]

BY ROSWELL PARK, A. M., M. D.,
Professor of surgery, Medical Department of the University of Buffalo.

LIKE a huge and frowning sphinx at the very gateway or entrance to the field of surgical pathology, has stood for centuries the great problem of the nature of cancer. This has at least until recently remained the inscrutable mystery of ages, and like the great sphinx at Ghizeh, it has been always a target for shafts of all kinds. Almost every theory which the ingenuity of the human race could conceive has been advanced to account for the existence of cancer, while each theory has been ardently supported until proven unsatisfactory, or often much longer. Today, while we desire always to pay due deference to the scholars and thinkers of time past, the only hypotheses which are even worth mentioning in this connection can be characterised as the dietetic, the embryological, the irritation, and the parasitic theories. The dietetic theory is of importance only in case we may succeed in maintaining the parasitic nature of the disease, when it may become a subsidiary question as to whether certain raw or uncooked food may furnish at least some of the parasites in question. So far as can be seen, only to this extent can the matter of diet have anything to do with the nature of cancer. How far it may come into consideration in explaining rapidity of growth, or even susceptibility to infection, is another matter, also of subsidiary importance.

The embryological theory of Cohnheim was a vast stride in advance, in that it offered the explanation of many curious and anomalous growths, but nothing contained within that hypothesis can

1. Abstract of a paper read before the Surgical Section of the American Medical Association, at St. Paul, June 5, 1901.

ever explain the peculiar behavior of cells which constitute the essential features of malignant growths. Cohnheim's explanation is sufficient to account for the presence of certain cells in unusual localities, but not for their peculiar behavior. The relation of irritation and trauma to cancer formation has always attracted attention, and today figures perhaps as prominently as it ever did in this respect. That malignant growths frequently do follow local injuries and local irritations is most certain. The same thing is exactly as true of certain of the infectious granulomata, especially of tubercular lesions, and viewed in its proper aspect we must hold that, while serious injuries provoke extensive and serious reaction, minor injuries are often followed by such incomplete reaction and such altered tissue relations as to leave points of least resistance, which in tuberculosis, as in cancer, are favorite foci for infection. Granting the ubiquitousness of the infectious agent, and a point of lessened resistance, one may easily appreciate the relation of trauma to cancer. The same is true of that constant but trifling traumatism which we term local irritation, as, for instance, from a dirty pipe or a jagged tooth.

The parasitic or infectious theory of cancer is the only one which satisfies the needs of both the pathologist and of the clinician. There has always been a strange apathy among the clinicians who meet so often the antemortem features of this disease, but who apparently remain so aghast at its mysteries that they have been content to leave its solution to the microscopist. The histologists, on the other hand, have their only opportunity of studying the disease in dry and hardened sections beneath the lens. The viewpoints of these respective gentlemen have been so widely different that they have rarely, if ever, gotten together and discussed the situation on common ground. All examinations of fresh and still warm specimens and all interpretation of the phenomena during life, have been neglected in this too minute division of labor. It was the realisation of all this that impelled me years ago to outline the character of the investigation required, and to secure from the legislature of the State of New York the facilities for furthering it.

Today, you will with me hold this to be true, that the history of absolute failure during time past to demonstrate an intrinsic cause, combined with accurate clinical observation and constant contact with cases, with a study of the disease as it occasionally occurs in endemic form, with the argument by analogy— upon which I shall elaborate in a moment— and with recent microscopic studies, all conspire to stamp the disease as due to an extrinsic cause, *i.e.*, to be parasitic in origin. Thus far one may safely go without transgressing well defined limits. Are we now able to go further and say that the parasites have been discovered? For myself, I do not hesitate to answer this positively in the affirmative; that is, I am firmly of this opinion, based upon work done elsewhere, and especially upon that done, to my own positive knowledge, in our own laboratory, and corroborated apparently by the independent investigations of Pfeiffer, Sawtschenko, Sjöbring, Eisen, and most recently, of Max Schüler, whose new monograph just published is the most striking, independent, and beautifully satisfactory demonstration of every statement that has emanated from the Buffalo laboratory that can be imagined.

The arguments that can be adduced in favor of the parasitic hypothesis can be summarised in a few pages. For this purpose, first, take the argument by analogy, beginning with the lower forms of life. Tumors in trees and plants are well known to vegetable pathologists and botanists. They occur so often as to escape notice. They vary in size from the most trifling gall to those large woody masses known as xylomata, which bring about the eventual destruction of the tree. They are frequently spoken of as tree cancers. These are due almost invariably to a species of infection. In other instances the first injury is traumatism, or its equivalent, such as is produced by the freezing of water contained within the grooves of the bark and the consequent splitting open of this protective covering, with its possibility for infection of the growing wood beneath. Through this laceration infectious agents enter from without and produce similar results. When carried to the extreme the nutrition of the limb, or the entire trunk, may be so far disturbed as to determine the death of the whole, or a part, of the tree. Were parasitic agents entirely excluded, there would be no such thing as cancer of trees. Could they be excluded, every laceration of bark would heal, as does any protected wound in animal forms, and the result would be protection from irritation and normal repair. The more one studies tumor formation in the vegetable kingdom, the more striking will the analogy become.

Comparative pathology will furnish many other arguments. Tumors are common in the lower animal forms and would be found much more so, if our observations were more minute and exact. One cannot avoid the conclusion that tumors in animals and man are due to the same general causes. If, then, their existence in animals can be proven to be of parasitic origin, it strengthens the conclusions in favor of a similar origin for such lesions in man.

One of the strongest of all arguments, and one which is really irrefutable, is that which we may get from the study of well-known infections which produce tumor formations. Take, for instance, the so-called infections granulomata. The essential differences between these and cancers are not their histological structure, but the fact that we know their minute causes. These tumors which used to be included with malignant tumors have been put in a class by themselves, simply because their causes are now well known, and modern writers have drawn very sharp lines between these tumors about which they have learned much and cancers about which they know very little. And yet these infectious granulomata are in many instances just as fatal as are the true cancers, and are amenable to the same methods of treatment and permit of the same general management; *i. e.*, they are distinctly surgical lesions.

Take, again, the matter of metastasis. There is no known infectious disease characterised by metastasis, from the most acute of the septic or pyemic type to the slowest manifestations of tuberculosis, in which we do not regard metastatic lesions as one of the principal evidences in favor of their infectiousness. Why pathologists have been so loath to see in similar manifestations of cancer a like evidence of its own infectiousness, I have never been able to understand.

Take, again, the matter of the local infectivity of cancerous lesions. The numerous repeated and now well known instances in which cancerous infection has followed the track of such instruments as the trocar, for example, afford other evidences whose value is undeniable. Aside from anything that the microscope may show, a few cases of this kind will have more value than all the failures of all the experimenters of the past century.

Regarding the microscopical appearances of these growths, we have had almost numberless hypotheses advanced to account for the well-known fact, that in and between the cells of cancerous growths are seen peculiar forms or particles which have been regarded by some as parasites, by others as products of cell degeneration, and by others yet as pure artefacts. It has been hard for observers to prove that they are cell degenerations and there are wide differences of opinion between those who hold to this view, as well as among those who regard them as parasites. Certain it is that no such appearances are noticed in healthy tissues, or in the infectious granulomata, or in the truly benign tumor. They must be either cell degenerations or parasites. No one has ever been able to reproduce such degenerations under other circumstances, nor are they known or scarcely even named. On the other hand, exactly similar appearances have been produced in large numbers after inoculation or experimentation.

It is true that observers have differed widely regarding their parasitic nature. Their exact nature is of secondary importance if their parasitic rôle can only be established. The first thing to prove is that cancer is of parasitic origin. Can this be done? It has been done, for instance, in the case of malaria where the parasites are seen inside as well as outside of the blood corpuscles, and, although we as yet know little, if anything, about their biological characteristics, we nevertheless accept them as parasites. Exactly similar appearances can be noted in and around the cells of rapidly growing cancers, and especially in parts where growth is most recent and rapid. Moreover, if scrapings be taken from a cancer while still fresh and warm, there may be observed under the lens unmistakable ameboid movements of many of these bodies which, when stained in sections, are seen in locations mentioned above.

This is not a question of bacteria. It is now a question of organisms, about which as yet we know very little; as little, in fact, as the profession in general knew of bacteria when Cohn first began studying them. We turn then from bacteria to some other form of parasitic life as the necessary explanation of known phenomena. Herein lies our greatest difficulty. We are as yet almost in total ignorance of these lowly forms of life and their biological peculiarities. We do not even know that Koch's laws for the determination of the infectious nature of a given disease are valid here, for these forms, since they may not fully apply to such varied conditions. Nevertheless, we may still hold to them until they are proven invalid.

For that matter, we have almost complied with these canons in our Buffalo investigations. In almost every instance we have found organisms in cancer cases; and in practically every instance by the introduction of cultures made from these organisms we have produced fatal results in animals, although we cannot truthfully say that in every instance we have produced distinct carcinomata. We do not yet know that this can be done in animals by the forms which produce it in man. We know that syphilis and various other diseases are not communicable to animals, and in the beginning of this investigation we must not be too exacting until certain other conditions are also complied with. Where we have so far failed in complying with Koch's canons lies especially in this direction—that we are unable yet to say that wherever we find the organism we find the disease. We do not yet know the cancer organism well enough to be able to identify it outside the human body, and, therefore, while this requirement has not yet been complied with, neither has it been constantly violated.

I have often heard it adduced as an argument against the parasitic theory, that the duration of cancer is altogether too long to permit its recognition as an infectious disease. Such statements as these have nothing to justify them. I may be pardoned foi calling attention to this fact which I have stated before, because of its importance in this connection. Infections follow no known limit or time law. There are some which are exceedingly acute, like cholera, bubonic plague, etc.; they kill in a few hours. There are others which run their course in a few days, like pneumonia, tetanus, diphtheria, etc. Still others extend over a period of a few weeks, like typhoid fever and glanders. The time in yet others is only measured by months. This is true of actinomycosis and of tuberculosis, whereas some instances of the latter disease and syphilis extend over years. No matter how long their course may be, we do not decline to see evidences of infection therein, and so the argument against the infectious nature of cancer because it lasts sometimes so long must fail. Cancer certainly is often as rapid as tuberculosis, even of the acute miliary form, and is usually more rapid than are syphilis or leprosy.

But, some one may ask, what about the failure of numerous experimenters to reproduce the disease by inoculation, or by implantation? I grant quite readily that until very recent times there has been almost complete disappointment in efforts in this direction. The disappointment, however, has not been so comprehensive as in the case of syphilis, since every effort in this direction has failed, and yet we do not deny the infectiousness of syphilis on that account. Because of the paucity of successes in this experimental work there attaches a certain suspicion or uncertainty to every alleged successful experiment. For me, however, as remarked above, one instance of cancer following the use of an instrument along the track which it has made is of very great value, far greater indeed than, on the other hand, would attach to 100 failures at deliberate implantation. Failure does not prove that success may not be finally attained, whereas one positive success is indisputable. Surely, clinical observation has amply established the possibility of infection with cancer. Once that be granted, the principal contention is maintained. Failures in times past have been largely due to our ignorance regarding the biology of these minute organisms, and the conditions which favor their life or death. In our laboratory work, for instance, in Buffalo, it has been discovered that they grow best in an exceedingly weak solution, and that ordinary bacteriological methods and culture media are absolutely inadequate. We have had best results in culture experiments by the use of an expedient suggested by Drs. Gaylord and Clewes in the use of collodion sacs. A little capsule is made of collodion; this is filled with cultures or with fresh fluids; it is sealed with collodion, then dropped into the peritoneal cavity of an animal. The organisms cannot escape through the collodion, but there is sufficient osmosis of the living fluids surrounding the sac to permit them to grow, under natural conditions, and they thus undoubtedly grow in the living animal without possibility of escape. Under these circumstances, their virulence is very much enhanced, and it is found that of its contents after removal of this sac from the first animal and inoculation into the second animal, they will produce a very rapid hematogenous infection, with nodular formation in various parts of the body, corresponding exactly to acute miliary carcinosis, the nodules thus produced often having the minute character of adenocarcinoma.

It certainly is not too strong a statement, then, if I claim that in the Buffalo laboratory Dr. Gaylord and our staff have absolutely produced adenocarcinoma by inoculation in a number of animals, and that this can now be produced in such a way as to afford unmistakable evidence of the infectivity of the disease.

When asked for a minute description of these organisms, it can scarcely yet be given. It must be enough for the present to say that they appear to belong to the protozoa, or possibly, as Schüler has hinted, to some still lower or less known animal form. They can be seen to undergo ameboid movement upon the warm stage, and careful study will reveal numerous changes which they undergo somewhat slowly, by which very positive and somewhat remarkable alterations in size, shape and general appearance are brought about. This would appear to take them out of the realm of the vegetable kingdom, and put them in that of the animal kingdom, and for the present it is enough to call them protozoa. Minute description or detailed statements can hardly be made yet, since they have to be most carefully studied, and, in fact, the whole matter of the biology of these lowly forms of animal life has to be gone over again before one talks too much about them.

There seems to be the most absolute resemblance between results obtained in the Buffalo laboratory and those just published by Max Schüler. There is scarcely a statement which he makes which is not corroborated by our own experiments. It seems to me that the statements of Schüler take away almost the last element of doubt which can remain as to the propriety of our conclusions regarding the parasitic nature of cancer.

Of course, it is yet too soon to formulate any conclusions regarding its treatment or therapy. For the present, at least, cancer must remain, as it always has been, a surgical disease. I believe this general statement can be made, that if cancer can be recognised early in accessible parts of the body and be removed thoroughly, it can be absolutely cured. Unfortunately, early recognition is rare, and thorough removal too infrequent. Consequently, we have the present hideous picture of the disease displayed before the profession. For cancer in inaccessible parts of the body, diagnosis must necessarily be late, and operative treatment can benefit little, if at all. If, however, we can establish a parasitic cause and cultivate a sufficient acquaintance with the organisms at fault, it is not too much to hope that some agent, be it vegetable or mineral drug, or animal antitoxin, may yet be discovered by which the ravages of the disease may be checked or prevented. Drugs are known which destroy the protozoa that cause malaria. Let us hope that something may yet be found, and that speedily, which may have the same destructive effect upon the parasites which produce cancer, without being inimical to the animal cells of the human body. Until this can be brought about, cancer is still a surgical disease.

